# Survival and behavior of free and encapsulated probiotic bacteria under simulated human gastrointestinal and technological conditions

**DOI:** 10.1002/fsn3.1531

**Published:** 2020-04-17

**Authors:** Muhammad Zeashan, Muhammad Afzaal, Farhan Saeed, Aftab Ahmed, Tabussam Tufail, Awais Ahmed, Faqir Muhammad Anjum

**Affiliations:** ^1^ Institute of Home & Food Sciences Government College University Faisalabad Pakistan; ^2^ The University of Gambia Gambia Gambia

**Keywords:** dessert, encapsulation, probiotics, stability, survival, wall materials

## Abstract

The present study was designed with the objective to compare the viability and stability of free and encapsulated probiotics under simulated technological and human gastrointestinal conditions. *L. acidophilus* was encapsulated using two wall materials (sodium alginate, soy protein isolate, and SA‐SPI) by extrusion method for enhanced viability under stressed conditions. Free and encapsulated probiotics were subjected to some simulated technological and gastrointestinal conditions. Furthermore, free and encapsulated probiotics were also incorporated in dairy dessert to evaluate the viability and stability during storage. Encapsulation using sodium alginate and SPI as a coating materials significantly (*p* < .05) improved the survival of probiotics under simulated gastrointestinal and thermal conditions. The buffering effect of microbeads prolonged their survival and stability of under simulated conditions. The number of surviving probiotic cells encapsulated with sodium alginate, SPI, and SA‐SPI over 120 days of product storage was 7.85 ± 0.39, 7.45 ± 0.37, and 8.50 ± 0.43 cfu/ml, respectively. In case of free cells, the surviving cells were just 3.5 ± 0.18 cfu/ml over the period of storage. In short, the study depicted that encapsulation provides protection during exposure to various hostile conditions.

## Graphical abstract

The comparison of the viability and stability of free and encapsulated probiotics under simulated technological and human gastrointestinal conditions. Purposely, the *L. acidophilus* was encapsulated using three biopolymer/wall materials (sodium alginate, soy protein isolate, and SA‐SPI) by extrusion method. Obtained microbeads and free probiotics were subjected to adverse technological and gastrointestinal tract conditions.

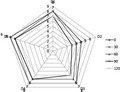

## 1 INTRODUCTION

Probiotics are described as “live beneficial micro‐organisms that, when ingested in sufficient quantities boost up host's immunity against intestinal pathogens and prevent an array of gastrointestinal disorders” (Hill et al., 2014). Various compounds like organic acids (lactic and acetic acids), produced by probiotics bacteria decrease the pH of growth medium thereby inhibiting the pathogen's growth. *Lactobacilli* represent a substantial part of intestinal microflora, and their relationship with the general state of human health is still under rigorous investigation phase. The genus *Lactobacillus* is one of the major groups of lactic acid bacteria used in food fermentation and is thus of great economic importance. (Pyar & Peh, 2014).

In a wide range of food and beverage products such as fruit juices, yoghurt and sour milk probiotics exhibit plentiful health benefits to the human such as improving intestinal microbial stability, by producing antimicrobial substances inhibiting pathogenic growth, simulating and modulating the innate immune systems, exhibiting antimutagenic activities, and preventing carcinogenesis. The genera *Lactobacillus* and *Bifidobacterium* are the most important probiotic micro‐organisms commonly related with gastrointestinal tract. Probiotics used in different products should optimally accomplish all of the following measures: remain viable during industrial production and processes; retain viable under harsh storage conditions as well as survive in host gastrointestinal environment to deliver the actual health benefits to the consumer. However, most of the probiotics incorporated in food and beverage are sensitive to processing and environmental factors including low pH and heat. Stability and viability of probiotics during processing and gastrointestinal transit (GIT) can be improved by encapsulation (Praepanitchai, Noomhorm, & Anal, 2019).

Encapsulation of probiotics controls the discharge of active molecules and improves the organism viability by resisting the unfavorable conditions like variation in pH, moisture, and oxygen availability. (Dubey, Shami, & Bhasker, 2009). The main aim of employing encapsulation on commercial basis in food applications is to enhance the probiotic stability to improve the bioavailability and functionality (Milanovic et al., 2010; Shi et al., 2013). Sodium alginate (SA), is being extensively used for the encapsulation of probiotics due to its excellent pH‐responsive properties (Feng et al., 2020). Probiotics are highly sensitive to the food handling operations, digestive enzymes, pH, and mechanical strain in the stomach. Encapsulation can cowl the bitter taste of a few meals by means of inhibiting reactions with different additives, such as water and oxygen in adverse conditions (Nedovic, Kalusevic, Manojlovic, Levic, & Bugarski, 2011; Rescigno, Rotta, Valzasina, & Riccardi‐Castagnoli, 2001).

Ice cream is a frozen dessert that comprises of air cells scattered in a watery framework (Muse & Hartel, 2004). The three basic principle parts of frozen ice cream are air cells, ice crystals, and fat globules, which are dispersed in a continuous aqueous phase (serum). The uniform air distribution is a key factor in deciding the product melting resistance and mouth feel. Fermented food products have been extensively utilized as probiotic carrier, but the current study has been exclusively designed to probe the effect of encapsulation on the viability and stability of probiotics in nonfermented frozen desserts and under simulated conditions.

## 2 MATERIAL AND METHODS

### 2.1 Procurements

Probiotic *Lactobacillus acidophilus* (ATCC‐ 4356) was obtained from NIFSAT, University of Agriculture Faisalabad Pakistan. Milk was purchased from local dairy farm. Food additives, media, wall materials (sodium alginate and soy protein isolate), ringer solution, sodium chloride, hydrochloric acid, distilled water, calcium chloride, and porcine bile extract (Sigma‐Aldrich) were purchased from local scientific market.

### 2.2 Culture activation

Probiotic cells were activated by inoculating them in MRS (Man Rogosa Sharpe) broth at 37°C for 24 hr. Afterward, the cells were harvested by centrifugation (Thermo Scientific Megafuge 8R) at 1,960 *g* for 10 min at 4°C. The obtained beads were washed twice using phosphate buffer (pH 7.0).

### 2.3 Encapsulation of probiotic bacteria

*Lactobacillus acidophilus* was encapsulated by the method as described by (Gul & Dervisoglu, 2017). Briefly, solution of sodium alginate (2%), soy protein isolate (2%), and sodium alginate–soy protein isolate (1:1) % w/w was dissolved in distilled water. The prepared solutions were sterilized in autoclave (121°C for 15 min). After cooling, prepared solutions were mixed with culture (10^10^log cfu/ml) suspended in 0.1% sterile peptone at 9:1 (v/v) ratio. The encapsulation plan is shown in Table 1 **.** For extrusion method, obtained mixtures were homogenized with ultra‐turret at 1,960 *g* for 2 min and the suspensions were injected drop wise through a syringe into 0.2 M CaCl_2_ solution with gentle stirring. The formed hydrogel beads were shaken at 300 rpm for 30 min in CaCl_2_ for hardening. They were then filtered with sterile filter, washed twice with sterile distilled water, kept in sterile Petri dishes, and stored at 4°C.

**Table 1:** Cell type and encapsulation plan

Cell type	Description
C1	Free Cells (nonencapsulated)
C2	Encapsulated with Sodium Alginate (SA)
C3	Encapsulated with Soy Protein Isolate (SP1)
C4	Encapsulated with Sodium Alginate–Soy Protein Isolate(SA‐SPI)

### 2.4 Measurement of bead size and morphology of beads

The shape of the capsules obtained by extrusion method was observed using an optical microscope equipped with a digital camera. The size of capsules obtained by extrusion was measured using a digital micrometer.

### 2.5 Encapsulation efficiency

The encapsulation efficiency (EE %) expresses the efficiency of entrapped and survival of viable cells during microencapsulation procedure. The EE (%) was determined by digestion method as described by Afzaal, Khan, et al. (2019), Afzaal, Saeed, Arshad, et al. (2019), Afzaal, Saeed, Saeed, et al. (2019) with slight modifications.

The EE (%) for the probiotic bacteria was calculated as follows:

$$\text{EE\textbackslash{}\%}\;=\frac{{\text{Log}}_{10}N}{{\text{Log}}_{10}N_{0}}\times 100$$

where *N* is the number of viable cells entrapped in capsules, and *N*_0_ is the total number of cells added in solution.

### 2.6 Survival of free and encapsulated probiotics under heat treatment

The stability and viability of *Lactobacillus acidophilus*‐encapsulated beads were exposed to heat treatment by following the method described by Fang et al. (2012) with slight modifications. *Lactobacillus acidophilus‐*encapsulated beads (10^10^log cfu/ml) and free cells were placed in test tubes containing 9 ml of ringer solution. The test tubes were further incubated in a water bath at various temperatures (72, 63, and 50ºC) for 2 min. After incubation, samples were collected at different intervals. The samples were cooled downward to room temperature. The stability and viability of the free and encapsulated *Lactobacillus acidophilus* probiotics were assessed by Standard plate count.

### 2.7 Survival and stability of free and encapsulated probiotics under acidic conditions

Under acidic conditions, the survival of the *Lactobacillus acidophilus* probiotics encapsulated in the beads was evaluated through the protocol earlier described by Praepanitchai et al. (2019) with slight modification. The viability of encapsulated and nonencapsulated *Lactobacillus acidophilus* probiotics under acidic environment was evaluated at pH 6.5, 3.0, and 2.0. *Lactobacillus acidophilus* as free cell and encapsulated were added to test tubes containing 9 ml of MRS medium adjusted to the preferred pH with 5 M HCl or 1 M NaOH). Incubation was done at 37ºC for 3 hr, and then centrifugation of samples at 1,960 *g* for 10 min at 4ºC was carried out. The viability of the free and encapsulated *Lactobacillus acidophilus* probiotics was assessed by standard plate count method.

### 2.8 In vitro Gastrointestinal assay

Free and encapsulated cells in simulated gastrointestinal conditions were evaluated by method described by Afzaal, Khan, et al. (2019), Afzaal, Saeed, Arshad, et al. (2019), Afzaal, Saeed, Saeed, et al. (2019). The simulated gastric juice (SGJ) was prepared using the regents (sodium chloride, 0.5%, KCl, 0.2%, pepsin 0.3%, NaHCO3, 0.1%, and 0.022% (w/v) CaCl_2_) and the desired pH (2.5) was adjusted with (0.1M) HCl. Free and encapsulated cells of sodium alginate, soy protein isolate, and sodium alginate–soy protein isolate were added to the test tubes and incubated at 37°C. The viability of free and encapsulated cells was recorded at 0, 25, 50, 75, 100 min. Simulated intestinal juice was prepared with (pancreatic, NaCl, KCl, NaHCO_3_, and bile salts) and required pH was adjusted to 7.5. Similarly, the cell survival and stability in simulated intestinal conditions was enumerated.

### 2.9 Product development

The ice cream was prepared by following a standard recipe containing 11% fat, 12.5% nonfat solids, 14.5% sugar, mango flavor and color, 0.4% emulsifier and stabilizers, and 0.2% starch. The treatment plan for preparation of dairy dessert is given in Table 2. The total solids in the end product were 38%. The milk and milk cream were added to mixing tank, and the temperature was increased to 50 ºC. Skim milk powder, starch, sugar, emulsifier, and stabilizers were mixed at 40°C. Milk and glucose were added at 50ºC. After mixing of all ingredients, homogenization was carried out at 65 ºC and 150 bar pressure, and the ice cream mix was then pasteurization at 80°C for 15 s. Probiotic bacteria (free and encapsulated) were incorporated into the ice cream, and incubation was done at 40°C. The detailed treatment plan for the product development is shown in Table 2. The ice cream mix was then cooled to 4°C and stored in storage tank for 08 hr. During aging, color, flavor, and free and encapsulated bacteria were incorporated according to the treatment plan in the ice cream mix. The freezing of ice cream was carried out in two stages, that is, dynamic freezing and static freezing. In dynamic freezing, the ice cream mix was frozen quickly while being agitated to incorporate air and to limit the size of ice crystals formed while in static freezing the partially frozen product after pouring in cardboard cups was hardened without agitation at −20°C. (Zanjani, Ehsani, GhiassiTarzi, & Sharifan, 2018).

**Table 2:** Product development plan

Desert type	Description
D1	Dessert without Probiotic Bacteria (Control)
D2	Dessert added of Free Cell
D3	Dessert added of Sodium Alginate Beads (SA)
D4	Dessert added of Soy Protein Isolate Beads (SP1)
D5	Dessert added of Sodium Alginate –Soy Protein Isolate (SA‐SPI)

### 2.10 Enumeration of probiotics in ice cream during storage

The survival of entrapped and free cells counts in ice cream during storage was determined by method as described by (Gul & Dervisoglu, 2017). Briefly, 1 g of each sample was weighted I and poured in tube containing 9 ml of ringer solution preheated to 37°C and then dropped into sterile stomacher bag. After homogenization for 15 min, 1 ml of homogenate sample was serially diluted with 9 ml of ringer solution and samples were plated on MRS agar. The plates were incubated under anaerobic conditions at 37°C for 48 hr. All enumerating plates of probiotics were incubated at 37°C for 3 days, and the results were recorded in colony‐forming units per g (cfu/g).

### 2.11 pH

The pH of the each sample of ice cream was determined using HANNA pH meter. The pH meter was calibrated using buffered solution of pH 4.00 and 7.00.

### 2.12 Sensory evaluation of ice cream

A panel of twenty expert judges of Government college university Faisalabad took part in the sensory evaluation of ice cream (Meilgaard, Civille, & Carr, 2007). The panelists evaluated the ice cream samples for the attributes like color, flavor, body, texture, and overall acceptability on 9‐point hedonic scale. The sensory analysis was carried out at an interval of 0, 30, 60, 90, and 120 days for all four treatments.

### 2.13 Statistical analysis

All the data were directly subjected to ANOVA (analysis of variance) to observe the significant difference (*p* < .05) between different treatments. The results were stated as the mean values from the three replicates.

## 3 RESULT AND DISCUSSION

### 3.1 Measurement of bead size

The polynomic polymer solution of sodium alginate and soy protein isolate in aqueous solution containing a suitable divalent counter cation like Ca^2+^ has the tendency to form hydrogel beads. Accomplishing smooth and more spherical microbeads can be obtained by enhancing the total solid content as earlier reported (Zaeim, Sarabi‐Jamab, Ghorani, Kadkhodaee, & Tromp, 2018). Size of the microbead is also affected by the viscosity of the hydrogel materials (Kruif & Tuinier, 2001; Maltais, Remondetto, & Subirade, 2008). The results regarding the bead size of various types of encapsulated probiotics are given in Table 3 **.** The result indicated that maximum bead size was found in C4 (Encapsulated with Sodium Alginate–Soy Protein Isolate), followed by C3 (Encapsulated with Soy Protein Isolate), and C2 (Encapsulated with Sodium Alginate). The maximum bead size in C4 (Encapsulated with Sodium Alginate–Soy Protein Isolate) could be due to encapsulating material because when sodium alginate and soy protein isolate combine together then viscosity of solution effect the size. Similar observations have been reported by Praepanitchai et al., 2019 who stated that the type of encapsulation materials affect the bead size.

**Table 3:** Size of the microbeads

Type of cells	Bead size (mm)
C2	1.02 ± 0.05
C3	1.17 ± 0.03
C4	1.29 ± 0.06

Note

All the values are expressed as a mean is 03 value of *SD*.

### 3.2 Encapsulation efficiency

The encapsulation yield or the encapsulation efficiency is affected by the type of the hydrogel materials and the method used for encapsulation. The incorporation of prebiotics matrix can result in higher encapsulation efficiency (Soukoulis et al. 2014). Encapsulation efficiency of different hydrogel materials used in this study is shown in Table 4. The result indicated that maximum EE% was in found in case of C4 (Encapsulated with Sodium Alginate–Soy Protein Isolate) followed by C3 (Encapsulated with Soy Protein Isolate), and C2 (Encapsulated with Sodium Alginate).

**Table 4:** Encapsulation efficiency

Type of cells	Numbers before encapsulation	Numbers after encapsulation	Efficiency (%)
C2	8.39 ± 0.04	7.97 ± 0.06	95
C3	8.40 ± 0.05	8.06 ± 0.08	96
C4	8.73 ± 0.09	8.54 ± 0.03	98

Note

All the values are expressed as a mean is 03 value of *SD*.

### 3.3 Thermal stability of free and encapsulated probiotics

Probiotics must survive the recommended pasteurization temperatures to be useful and remain viable in food and beverage products. Survival of probiotic cells in free cells, encapsulated with sodium alginate, encapsulated with soy protein isolate, and encapsulated with sodium alginate–soy protein isolate hydrogel beads, was evaluated under different heat treatment at 72°C, 63°C, and 50°C and results shown in Table 5. In C2 (Encapsulated with Sodium Alginate), C3 (Encapsulated with Soy Protein Isolate), and C4 (Encapsulated with Sodium Alginate–Soy Protein Isolate) cells, significant viability up to 6.8 to 8.8 log cfu/ml. The probiotic in the C4 (Encapsulated with Sodium Alginate–Soy Protein Isolate) treatment beads prepared achieved the highest survival rate (8.90 ± 0.45log cfu/ml) after heat treatment at 50°C for 1 min. However, the survival of free probiotic was 4.50 ± 0.23 log cfu/ml which is not adequate to achieve maximum benefits of probiotics. Comparatively, the survival rate of probiotics cells encapsulated with SA and SPI was less than SA‐SPI. The results confirmed the presence of probiotic bacteria at minimum levels of 10^7^–10^8^ log cfu/ml, which is recommended therapeutic level in functional foods. Fang et al. (2012) reported that the cells encapsulated with SA‐SPI hydrogel beads provided better protection on the viability of *Lactobacillus acidophilus* more than free, SA‐, and SPI‐encapsulated cells. The result of the study is also in line with the experiments conducted by results were promising and corroborate with those obtained by Rather, Akhter, Masoodi, Gani, and Wani (2017) who reported that encapsulation maintain higher cell count during exposure to heat treatment.

**Table 5:** Thermal stability of free and encapsulated probiotics

Temperature			
Types of cells and survival (log_10_cfu)	50°C	63°C	72°C
C1	4.5 ± 0.23	4.3 ± 0.22	3.5 ± 0.18
C2	7.80 ± 0.39	7.40 ± 0.37	6.80 ± 0.34
C3	7.90 ± 0.71	7.60 ± 0.38	7.10 ± 0.36
C4	8.90 ± 0.45	8.85 ± 0.44	8.80 ± 0.44

Note

All the values are expressed as a mean is 03 value of *SD*

### 3.4 Evaluation of survival of encapsulated probiotics in acidic solutions

The data on the survival of encapsulated probiotics in acidic solution are demonstrated in the Table 6. It is shown that at pH 2, C1 (Free Cells) showed minimum viability while C2(Encapsulated with Sodium Alginate), C3(Encapsulated with Soy Protein Isolate), and C4 (Encapsulated with Sodium Alginate–Soy Protein Isolate) showed viability 6.10–7.75 log cfu/ml, but the maximum viability was shown by C4 (Encapsulated with Sodium Alginate–Soy Protein Isolate) 7.75 ± 0.39 cfu/ml. The same trend was found at pH 3 and 6.5. Maximum trend was found at pH 6.5 of C4 (Encapsulated with Sodium Alginate–Soy Protein Isolate), which was 8.6 ± 0.43 cfu/ml. At pH 6.5, the results of all four treatments were between 7.1 and 8.6 log cfu/ml because bacteria can survive better at pH 6.5, so total viable counts were observed. Soy protein isolate–sodium alginate combined seems to exert a synergetic effect on the survival of encapsulated probiotics. Ding and Shah (2009) also found that probiotics can survive better in encapsulation rather than free cells. Many other scientists have reported that the use of biopolymer prolongs the viability and stability under acidic conditions (Su et al., 2018).

**Table 6:** Survival of free and encapsulated probiotics

pH of acidic solution			
Types of cells and survival (log_10_cfu)	2	3	6.5
C1	2.30 ± 0.12	3.5 ± 0.18	7.1 ± 0.36
C2	6.1 ± 0.31	6.9 ± 0.35	7.8 ± 0.39
C3	6.15 ± 0.31	7.1 ± 0.36	7.9 ± 0.40
C4	7.75 ± 0.39	8.20 ± 0.41	8.6 ± 0.43

Note

All the values are expressed as a mean is 03 value of *SD*.

### 3.5 Viability and stability of encapsulated probiotics in simulated gastric conditions

The viability and stability of probiotic bacteria are very important in GIT. Feasibility of probiotic cells is vital in stomach and intestinal conditions so that the desired benefits of probiotics can be achieved. The probiotic cells (nonencapsulated and encapsulated) were subjected in gastric juice results are shown in Table 7. A rapid log reduction was observed for nonencapsulated bacteria in contrast to encapsulated probiotic cells. Encapsulation of SA‐SPI results better for the survival of probiotics as compared to SA and SPI as shown in Table 7. The results confirmed that encapsulation has a shielding effect toward probiotics in simulated gastric conditions. De Prisco, Maresca, Ongeng, & Mauriello, 2015 also found that in simulated gastric conditions probiotic survive better when encapsulated with different materials. Yasmin, Saeed, Pasha, and Zia (2019) found that the use of whey proteins as wall materials provided protection in various stressed conditions.

**Table 7:** Survival of free and encapsulated probiotics under gastric conditions

Time (Minutes)					
Types of cells and survival (log_10_cfu	0	25	50	75	100
C1	7.2 ± 0.36	6.50±0.33	5.2 ± 0.26	4.3 ± 0.22	3.5 ± 0.18
C2	7.75 ± 0.39	7.40 ± 0.37	7.35 ± 0.37	7.20 ± 0.36	7.05 ± 0.35
C3	7. 80 ± 0.39	7.50 ± 0.38	7.35 ± 0.37	7.20 ± 0.36	7.15 ± 0.36
C4	8.25 ± 0.41	8.20 ± 0.41	8.10 ± 0.41	7.90 ± 0.40	7.85 ± 0.39

Note

All the values are expressed as a mean is 03 value of *SD*.

### 3.6 Stability and viability of encapsulated probiotics in intestinal conditions

Wall materials that are dissimilar showed a shielding result on probiotics after they were exposed to the intestinal conditions. Current study showed probiotics in free (un‐encapsulated) and encapsulated form were added in artificial simulated intestinal solution for a defined time period. A sudden drop in probiotics which were without encapsulation was observed in contrast to the encapsulated cells at pH 7.5 as shown in Table 8. The C2 (Sodium Alginate), C3 (Soy Protein Isolate), and C4 (Encapsulated with Sodium Alginate–Soy Protein Isolate)cells showed a gentle log reduction in comparison with nonencapsulated probiotics as showed in Table 8. Current study results are in line with the findings of Afzaal, Khan, et al. (2019), Afzaal, Saeed, Arshad, et al. (2019), Afzaal, Saeed, Saeed, et al. (2019) who stated that encapsulation of cells with alginate enhanced the viability of probiotic in GIT environment. The results are also in line with Shi et al. (2013) who observed that encapsulation could improve the stability of probiotic bacteria in simulated gastrointestinal conditions.

**Table 8:** Survival of free and encapsulated probiotics under intestinal conditions

Time (Minutes)					
Types of cells and survival (log_10_cfu)	0	25	50	75	100
C1	7.25 ± 0.36	6.5 ± 0.33	5.75 ± 0.29	4.3 ± 0.22	3.5 ± 0.18
C2	7.75 ± 0.39	7.45 ± 0.37	7.35 ± 0.37	7.20 ± 0.36	7.05 ± 0.35
C3	7. 80 ± 0.39	7.50 ± 0.36	7.42 ± 0.37	7.23 ± 0.36	7.15 ± 0.36
C4	8.25 ± 0.41	8.18 ± 0.41	8.10 ± 0.41	7.99 ± 0.40	7.95 ± 0.40

Note

All the values are expressed as a mean is 03 value of *SD*.

### 3.7 Probiotic viability and stability in dessert during storage

The data on the total viable count in ice cream are demonstrated in the Table 9. Total viable probiotic were studied on storage for 120 days with intervals (0, 30, 60, 90, and 120). It was observed that during storage D2 (Dessert added of Free Cell) could not maintain the desired number of cells. The viable cell count in case of free cells decreased from 8.90 ± 0.45 to 3.5 ± 0.18 cfu/g, which is not adequate to attain desired health benefits. On the other hand, encapsulation with polymer and protein maintained recommended therapeutic level (10^7^–10^8^) during storage. Dessert samples, D5 (Dessert Encapsulated with Sodium Alginate–Soy Protein Isolate) more effective than D4 (Dessert added of Soy Protein Isolate (SP1) Beads) and D3 (Dessert added of Sodium Alginate (SA) Beads).Total viable count in D5 (Dessert added of Sodium Alginate –Soy Protein Isolate (SA‐SPI) Beads) just reduce to 8.50 ± 0.43 cfu/ml from 8.96 ± 0.45 cfu/ml with 120 days storage. Abghari, Sheikh‐Zeinoddin, Soleimanian‐Zad, (2011) reported that enlargement of ice crystals, occurring as a result of temperature fluctuations, affects the viability of the micro‐organisms in ice cream during storage. Sultana et al., 2000 reported that microencapsulation increased the survival of *Lactobacillus acidophilus* and *Bifidobacterium* as compared with free cells in frozen dairy desserts during stored. Yasmin et al. (2019) reported in their studies that the encapsulation of probiotics with different polymer significantly prolong the viability and stability during storage.

**Table 9:** Survival of free and encapsulated probiotics in desserts during storage

Storage (Days)					
Treatment (Survival of probiotics log_10_cfu)	0	30	60	90	120
D2	8.90 ± 0.45	6.5 ± 0.33	5.75 ± 0.29	4.3 ± 0.22 cfu/m	3.5 ± 0.18
D3	8.95 ± 0.45	8.75 ± 0.44	8.25 ± 0.41	8.05 ± 0.40	7.85 ± 0.39
D4	8.95 ± 0.45	8.77 ± 0.44	8.20 ± 0.41	7.90 ± 0.40	7.45 ± 0.37
D5	8.96 ± 0.45	8.85 ± 0.41	8.75 ± 0.44	8.60 ± 0.43	8.50 ± 0.43

Note

All the values are expressed as a mean is 03 value of *SD*.

### 3.8 pH

The result regarding the pH of ice cream with storage is given in Table 10. The result indicated that maximum pH was in found in case of D1 (Control Dessert) followed by D5 (Dessert Encapsulated with Sodium Alginate–Soy Protein Isolate), D4 (Dessert added of Soy Protein Isolate (SP1) Beads), D3 (Dessert added of Sodium Alginate (SA) Beads), and D2 (Dessert added of Free Cell). The pH of D2 decreased rapidly during storage that could be due to the activity of free cells. Zanjani et al. (2018) reported that during the storage, pH of the ice cream decreased which cause an increase in acidity due to the excessive sugar fermentation of milk sugar and the presence of lactic acid‐producing organisms during storage. The results of the study are also in line with the findings of Hekmat, & McMAHON, (1992) who reported that probiotics produce fast acid in ice cream mix.

**Table 10:** pH changes in desserts incorporated with free and encapsulated probiotics

pH changes during Storage (Days)					
Treatment	0	30	60	90	120
D1	6.60 ± 0.33	6.58 ± 0.32	6.57 ± 0.33	6.57 ± 0.33	6.56 ± 0.33
D2	6.45 ± 0.32	6.35 ± 0.32	6.30 ± 0.32	6.18 ± 0.31	6.09 ± 0.30
D3	6.54 ± 0.33	6.52 ± 0.33	6.50 ± 0.33	6.48 ± 0.32	6.45 ± 0.32
D4	6.53 ± 0.33	6.51 ± 0.33	6.50 ± 0.33	6.47 ± 0.32	6.45 ± 0.32
D5	6.55 ± 0.33	6.53 ± 0.33	6.52 ± 0.33	6.51 ± 0.33	6.49 ± 0.33

### 3.9 Sensory evaluation

**Figure 1:**
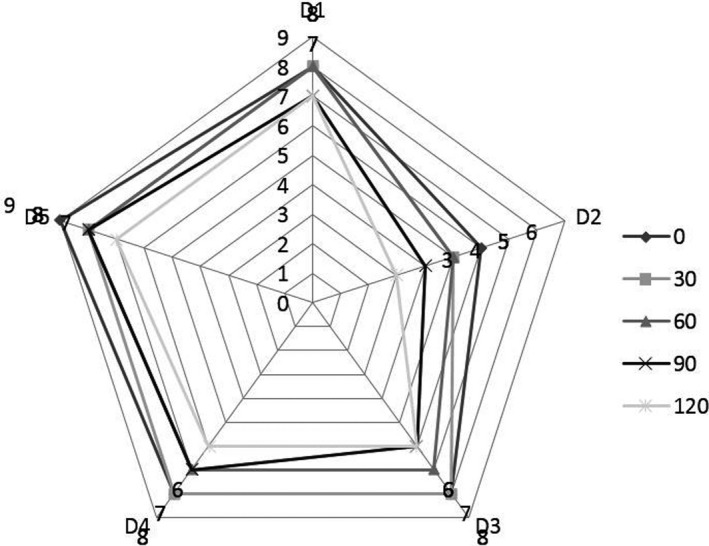
Sensory evaluation of desserts containing free and encapsulated probiotics

The consumer's response to product sensory evaluation is very important. The results regarding sensory evaluation are shown in Figure 1. Sensory evaluation score of D5 (Dessert added of Sodium Alginate–Soy Protein Isolate (SA‐SPI) Beads) was higher than all other treatments. Type of materials used for encapsulation directly affect the product texture, taste and overall acceptability. While D1 (Dessert without Probiotics) scored in acceptable range but less than D5 (Dessert added of Sodium Alginate –Soy Protein Isolate (SA‐SPI) Beads). However, ice cream with free cells observed very poor sensory evaluation of product with storage that is due to free cells produce more acidity and have poor texture. Gul et al. (2017) also found that dessert with encapsulation has exceptional sensory evaluation rather than dessert with free cells and without probiotic.

## 4 CONCLUSION

Encapsulating wall materials (sodium alginate, soy proteins isolate, and sodium alginate–soy proteins isolate) were found to be effective for augmenting the viability and stability of probiotics under different stressed conditions. Encapsulation with SA‐SPI combination showed best results in terms of encapsulation efficiency and viability. Encapsulated probiotic bacteria showed more thermal stability compared with free cells. Additionally, the incorporation of free and encapsulated probiotics affected the physiochemical and sensorial parameters of carrier food.

## CONFLICT OF INTEREST

The authors declare no conflict of interests.

## ETHICAL ISSUES

No ethical issues were promulgated.
